# Mapping occurrence of *Taenia solium* taeniosis/cysticercosis and areas at risk of porcine cysticercosis in Central America and the Caribbean basin

**DOI:** 10.1186/s13071-017-2362-7

**Published:** 2017-09-18

**Authors:** Uffe Christian Braae, Brecht Devleesschauwer, Fortune Sithole, Ziqi Wang, Arve Lee Willingham

**Affiliations:** 1 One Health Center for Zoonoses and Tropical Veterinary Medicine, Ross University School of Veterinary Medicine, Basseterre, West Indies Saint Kitts and Nevis; 2 Department of Public Health and Surveillance, Scientific Institute of Public Health (WIV-ISP), Brussels, Belgium; 3 Emerging Pathogens Institute and Department of Animal Health, University of Florida, Gainesville, FL USA; 4 Center for Translational Research in Neurodegenerative Disease, Department of Neuroscience, University of Florida, Gainesville, FL USA

**Keywords:** *Taenia solium* taeniosis/cysticercosis, Distribution, Mapping, Tapeworm, Neglected tropical disease

## Abstract

**Background:**

This study aimed to map the occurrence of *Taenia solium* taeniosis/cysticercosis at national level within Central America and the Caribbean basin, and to map the distribution of porcine cysticercosis at first-level administrative subdivision level (department level) and the porcine population at risk. This zoonotic parasite is believed to be widely endemic across most of Latin America. However, there is little information readily available for Central America and the Caribbean basin. *Taenia solium* has been ranked the most important foodborne parasitic hazard globally and within endemic areas is a common cause of preventable epilepsy.

**Methods:**

We conducted a structured literature search in PubMed, supplemented and crossed-referenced with relevant academic databases, grey literature, and active searches in identified literature, to identify all records of *T. solium* presence in Central America and the Caribbean basin between 1986 and April 2017. To retrieve grey literature, government entities, researchers and relevant institutions across the region were contacted in an attempt to cover all countries and territories. Identified records containing data on porcine cysticercosis were geo-referenced to identify department level distribution and compared to modelled distributions of pigs reared under extensive production systems.

**Results:**

We identified 51 records of *T. solium* at the national level, covering 13 countries and an additional three countries were included based on World Organisation for Animal Health (OIE) reports, giving a total of 16 countries out of 41 with evidence of the parasite’s presence. Screening records for porcine cysticercosis data at the departmental level confirmed porcine cysticercosis presence in 11 departments across six countries (Colombia, Guatemala, Honduras, Mexico, Nicaragua and Venezuela).

**Conclusions:**

When comparing these results to areas where pigs were kept in extensive production systems and areas where no information on porcine cysticercosis exists, it is apparent that porcine cysticercosis is likely to be underreported, and that a substantial part of the regional pig population could be at risk of contracting porcine cysticercosis. More detailed information on the distribution of *T. solium* and accurate burden estimations are urgently needed to grasp the true extent of this zoonotic parasite and the public health and agricultural problems it potentially poses.

**Electronic supplementary material:**

The online version of this article (10.1186/s13071-017-2362-7) contains supplementary material, which is available to authorized users.

## Background

The zoonotic tapeworm *Taenia solium* causing taeniosis in humans and cysticercosis in both pigs and humans is presumed to be widely distributed across areas of low- and middle-income countries where sanitation is insufficient and pigs are kept in extensive production systems. Pig herds in these systems consist of less than 10 pigs that are usually unconfined with scavenging supplemented with household waste [1]. Scavenging and household waste consumption can increase the risk of porcine cysticercosis transmission [2, 3]. Although classified as a major neglected tropical disease (NTD) by the World Health Organization [4], and recently ranked by two independent expert groups as the most important foodborne parasitic disease globally [5, 6], *T. solium* taeniosis/cysticercosis remains neglected in most endemic countries and overlooked as a potential public health problem in many other countries where the parasite could be present. The primary health burden of *T. solium* is the result of the clinical effects of neurocysticercosis, a common cause of preventable epilepsy in endemic countries [7], but the burden of cysticercosis is not exclusive to endemic countries. O’Keefe et al. [8] estimated based on nationwide inpatient records in 1998–2011, that the cysticercosis-related hospitalisations in the USA represented a rate of 8.03 per million people. O’Neal & Flecker [9] estimated that based on 18,584 hospitalisations for neurocysticercosis and associated hospital charges in 2003–2012 in USA, the total monetary burden was higher than USD $908 million, which constituted more than the costs for malaria or all other NTDs combined. As active transmission of *T. solium* is not presumed to occur to a great extent within USA, most of these cases were probably imported or the result of people contracting taeniosis abroad and then subsequently exposing Americans to risk [10], such as immigrants or travellers returning from endemic areas in e.g. Latin America where *T. solium* is endemic [11, 12]. In addition, the parasite causes a significant economic impact on agricultural sectors within endemic areas [13].

Bhalla et al. [14] conducted a comprehensive review of epilepsy incidence in South America and the Caribbean but found no data from the Caribbean. Most of the recent studies on *T. solium* from the Americas originate from either Mexico or Peru, but currently, there is no overview of the distribution of *T. solium* within Central America and the Caribbean basin. Specifically, there is no detailed distribution map of porcine cysticercosis and the porcine population at risk. Such maps could prove essential in decision-making processes regarding where intervention strategies should be implemented, and the underlying data could support the making of informed burden estimations across the region [15]. There are no current control programmes against *T. solium* implemented in Central America or the Caribbean basin, nor has there been in the past, except for smaller intervention studies [16, 17]. The aim of this study was (i) to map occurrence of *T. solium* taeniosis/cysticercosis at national level within Central America and the Caribbean basin, and (ii) map the distribution of porcine cysticercosis at a first-level administrative subdivision level (department level) and the porcine population at risk, based on presence of extensive pig production systems, which is a risk factor for the parasite.

## Methods

The study covered Central America and the Caribbean basin. The Caribbean basin is defined here as the Caribbean countries and territories in addition to Colombia, Venezuela, Guyana, Suriname, French Guiana, and the Yucatán Peninsula (Mexico). A list of countries and territories is available in Additional file 1: Table S1.

The data included in this study were (i) peer-reviewed studies of *T. solium* taeniosis/cysticercosis in Central America and the Caribbean basin, (ii) “grey literature” on *T. solium* taeniosis/cysticercosis presence in Central America and the Caribbean basin which consisted of informally published written materials such as reports and theses, (iii) modelled density of pigs kept in extensive production systems from 2006 [18, 19], and (iv) porcine cysticercosis reports from the World Organisation for Animal Health (OIE).

### Published and grey literature search

We performed a literature search using PubMed (http://www.ncbi.nlm.nih.gov/pubmed/) with a date restriction from 01 – 01–1986 to 12–04–2017 using the following search term: (solium OR Taeni* OR Neurocysticercosis OR Cysticerc* OR cellulosae) AND (Anguilla OR Antigua and Barbuda OR Aruba OR Bahamas OR Barbados OR Belize OR Bonaire OR British Virgin Islands OR Bermuda OR Cayman Islands OR Colombia OR Costa Rica OR Cuba OR Curaçao OR Dominica OR Dominican Republic OR El Salvador OR French Guiana OR Grenada OR Guadeloupe OR Guatemala OR Guyana OR Haiti OR Honduras OR Jamaica OR Martinique OR Mexico OR Montserrat OR Netherlands Antilles OR Nicaragua OR Panama OR Puerto Rico OR Saba OR Saint Kitts and Nevis OR Saint Lucia OR Saint Vincent and the Grenadines OR Saint Eustatius OR Sint Maarten OR Saint Martin OR Suriname OR Trinidad and Tobago OR Turks and Caicos Islands OR US Virgin Islands OR Venezuela). The same search term was used in Thomson Reuter’s Web of Knowledge (http://www.wokinfo.com). We also searched the following databases: LAMJOL (http://www.lamjol.info), SciELO.org (http://www.scielo.org), Cab Direct (http://www.cabdirect.org), and Société de Pathologie Exotique (http://www.pathexo.fr/) using the following keywords: “*taenia solium*”, “porcine cysticercosis”, “cysticercus cellulosae”, “neurocysticercosis”, “human cysticercosis”, “taeniasis”, “taeniosis”, “tenias”, “neurocisticercosis”, and “cysticercosis”. Hits found in any of the databases were cross-referenced with the initial search result from PubMed. References found were investigated for the presence of *T. solium* within Central America and the Caribbean basin to compile all known studies of the national level presence of the parasite. Studies from Mexico were only considered if data originated from the Yucatán Peninsula. Across the region, government representatives and research institutions dealing with livestock health or public health were contacted in efforts to retrieve any unpublished information on *T. solium* from their respective country or territory. See Additional file 1: Table S1 for a list of institutions and country representatives contacted.

**Fig. 1:**
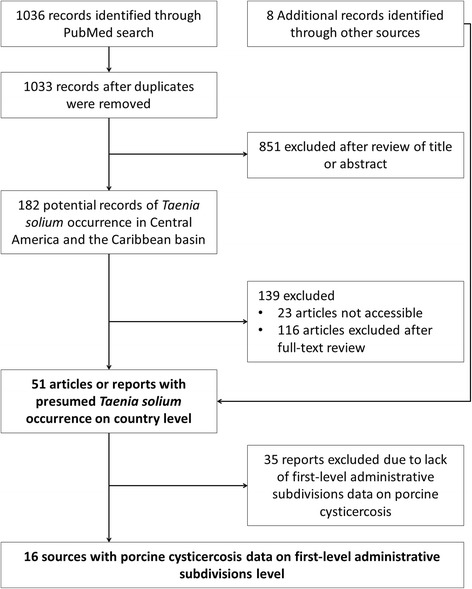
The search results using PubMed and additional resources

Initially, we reviewed all titles and abstracts for the occurrence of *T. solium* and excluded studies from outside the region. Full-texts of the remaining literature, if accessible, were scrutinised and excluded using the following criteria: not dealing with *T. solium,* no mention of geographical reference, experimental studies where no reference to geographical data of the parasitic material used was given, studies based solely on questionnaire surveys, and environmental studies. No language restrictions were imposed. All review articles were screened for potentially suitable references, as were all references in articles found to fit the inclusion criteria of this study. Authors of articles where full-text was inaccessible were contacted. Studies on human cysticercosis were only included if the authors provided the approximate location (country/territory) of where the patient was presumably infected. Studies on taeniosis where the infection was not confirmed to be *T. solium* taeniosis were not included in the overall national mapping unless human or porcine cysticercosis was also reported from that specific country. All studies identified with data on the occurrence of porcine cysticercosis at a national level were further investigated for department level geo-referenced data (Fig. 1).

### OIE data

Recent reports (2005 to 2016) of porcine cysticercosis presence were extracted from the OIE database WAHID Interface [20]. The database stores reports in six-month intervals with disease status divided into categories: disease present, limited distribution, suspected but not confirmed, not reported, never reported, and no information available. Only data for countries that had confirmation of porcine cysticercosis presence (i.e. “disease present” or “limited distribution”) were extracted.

### Modelled data

The density of pigs kept in extensive production systems was mapped using geographical information systems based on previously modelled data available through http://livestock.geo-wiki.org [18, 19].

## Results

The search strategy identified 51 records, in English, Dutch and Spanish, containing data supporting the presence of *T. solium* at the national level from 13 countries within Central America and the Caribbean basin (Table 1).

**Table 1:** Countries in Central America and the Caribbean basin with *Taenia solium* taeniosis/cysticercosis occurrence recorded from 1986 to April 2017. *Taenia solium* taeniosis was not confirmed unless otherwise stated and presence of *T. solium* there uncertain

Country	Porcine cysticercosis	Taeniosis	Human cysticercosis	No. of references
Belize			[31]	1
Colombia	[32–35]		[33, 34, 36–38]	7
Costa Rica			[39]	1
Cuba		[40]	[41]	2
Guadeloupe			[42]	1
Guatemala	[16, 43]	[16, 43–45]^a^	[43, 46]	5
Haiti		[47]	[48]	2
Honduras	[23, 49, 50]	[17, 49, 51–54]^a^	[17, 52–56]	11
Mexico^b^	[50, 57–59]	[59]	[59]	4
Nicaragua	[60]		[61, 62]	3
Panama			[63–65]	3
Saint Lucia		[29]^a, c^		1
Venezuela	[66–70]	[71–73]	[72–77]	12

^a^Confirmed *T. solium* taeniosis cases

^b^Only studies from the Yucatán Peninsula are included here

^c^Method used for confirmation not stated

**Fig. 2:**
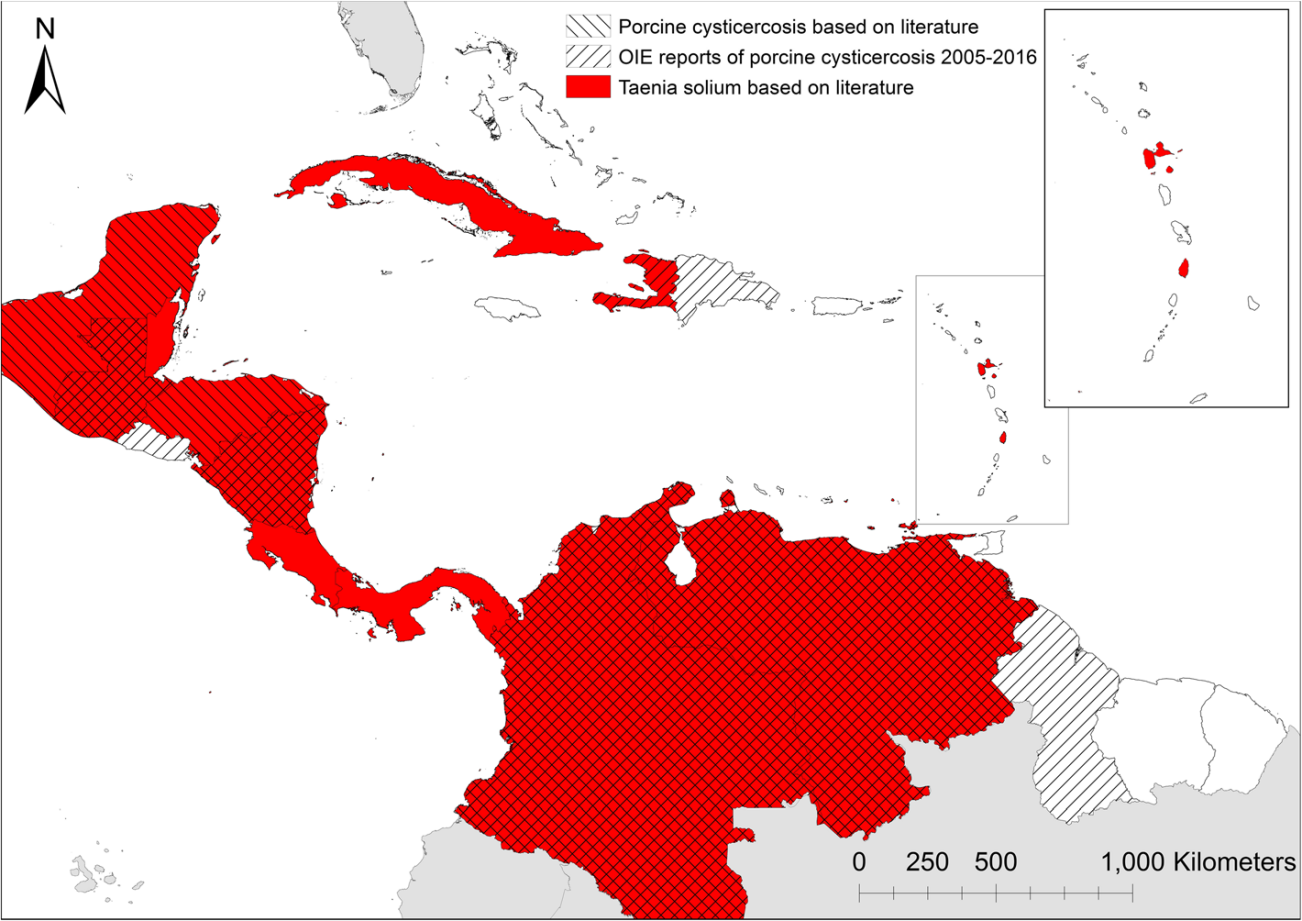
*Taenia solium* taeniosis/cysticercosis based on literature from 1986 to April 2017 and porcine cysticercosis based on OIE reports from 2005 to 2016 in Central America and the Caribbean basin

OIE reports from 2005 to 2016 additionally confirmed porcine cysticercosis presence in Haiti, the Dominican Republic, El Salvador and Guyana. Human cysticercosis, primarily reported as neurocysticercosis, was reported from all countries where *T. solium* was confirmed by literature with the exception of Saint Lucia where only taeniosis was reported. Taeniosis was reported from seven countries (Cuba, Guatemala, Haiti, Honduras, Mexico, Saint Lucia and Venezuela), but studies from Cuba, Haiti, and Venezuela were not confirmed to species level and can therefore not be ruled out as potential reports of *Taenia saginata* taeniosis. Overall, *T. solium* was confirmed in 16 countries out of 41 countries, and dependent territories within the region based on literature and OIE reports (Fig. 2).

**Fig. 3:**
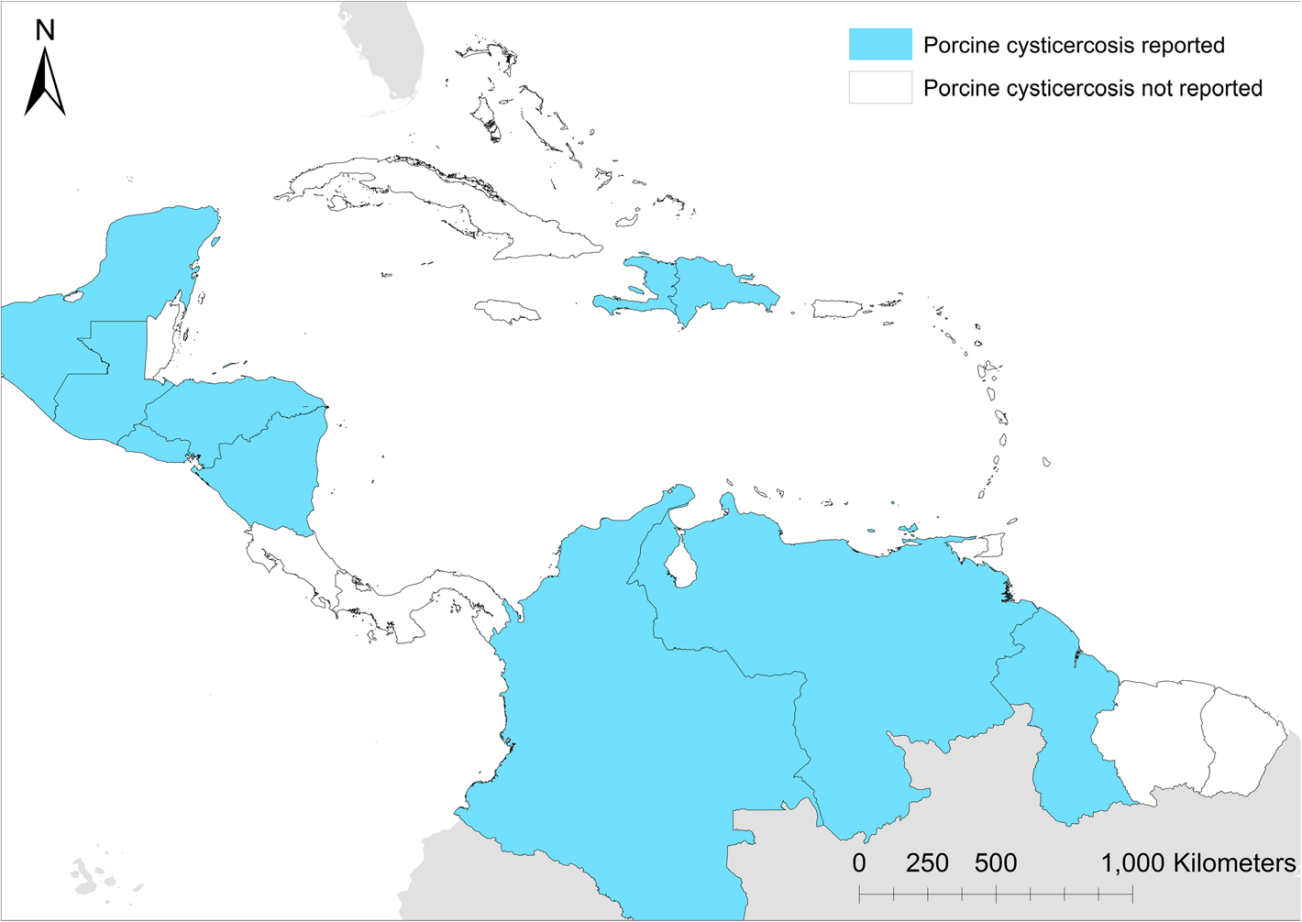
Endemic countries with reports of porcine cysticercosis in the period 1986–2016

**Fig. 4:**
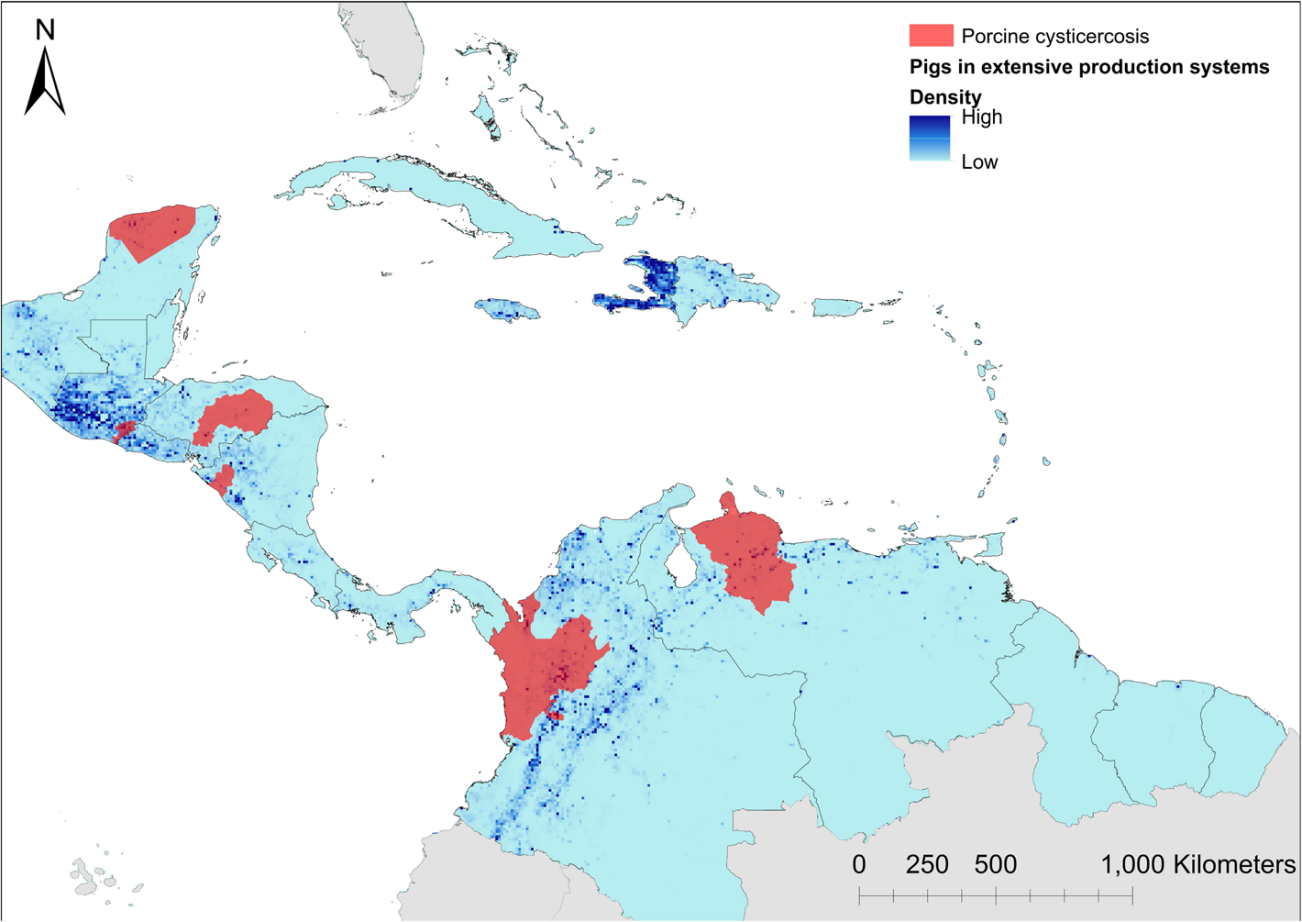
Departments (first-level administrative subdivision) with porcine cysticercosis in the period 1986 to April 2017. Density of pigs kept in extensive productions systems was extracted from [18, 19]

Porcine cysticercosis was confirmed from 10 countries through literature and OIE reports (Fig. 3). In all six countries (Colombia, Guatemala, Honduras, Mexico, Nicaragua and Venezuela) where porcine cysticercosis was identified based on literature, one (Nicaragua) based on unpublished data, data were also available to support presence of disease on a departmental level, but detailed distribution could not be retrieved for the additional four affected countries found through OIE reports. Porcine cysticercosis was reported in 11 departments from the six countries with departmental data (Table 2). Most reports were from Venezuela in four contiguous departments found to harbour *T. solium* infected pigs. Departments identified in Colombia and Honduras were also contiguous within each respective country. A single department was identified from Guatemala, Nicaragua, and the Mexican Peninsula of Yucatán, respectively. Figure 4 shows the departments found to harbour porcine cysticercosis infected pigs, overlaid with the previously modelled density of pigs kept in extensive production systems throughout the region [18, 19].

**Table 2:** The department (first-level administrative subdivision) level occurrence of porcine cysticercosis in Central America and the Caribbean basin from 1986 to April 2017

Country	Porcine cysticercosis	First-level administrative subdivision
Colombia	[33, 34]	Antioquia, Chocó
Guatemala	[16, 43]	Jutiapa
Honduras	[23, 49]	Francisco Morazán, Olancho
Mexico	[50, 57–59]	Yucatán
Nicaragua	[60]	Leon
Venezuela	[66–70]	Yaracuy, Cojedes, Falcon, Portuguesa

## Discussion

This study showed that *T. solium* taeniosis/cysticercosis was present in 16 out of the 41 countries and territories within Central America and the Caribbean basin. Porcine cysticercosis was identified in 10 of those countries (Fig. 3) and was confirmed at the department level in 11 departments from six of these 10 countries, covering six contiguous areas. These areas contain substantial pig populations [18], and with the exception of the Yucatán Peninsula (Mexico), also border areas with a relatively high density of pigs kept in extensive production systems. Figure 4 provides a visualisation of the pig populations at risk of porcine cysticercosis and areas at the departmental level with porcine cysticercosis. Based on the information provided here, *T. solium* could potentially be substantially underreported due to lack of epidemiological surveys conducted within the region.

The departmental level distribution map of porcine cysticercosis in Central America and the Caribbean basin provides a good overview of the known distribution of *T. solium* in pigs across the region. As illustrated in Fig. 4 the majority of the pig population kept in extensive production systems are outside areas identified to be endemic for porcine cysticercosis. This could be an indication of porcine cysticercosis not being a widespread problem, or that porcine cysticercosis is highly underreported. Surveys are needed to establish whether porcine cysticercosis is in fact only focally distributed within the identified countries and within the departments found to be endemic, or whether the distribution of porcine cysticercosis could be more widespread across the entire region. All areas where pigs are kept in extensive systems could potentially be at risk of porcine cysticercosis as this is a known risk factor for *T. solium* infection [2, 21], as could areas where lack of awareness on pig management standards and levels of sanitation are low [3, 22]. The endemic area identified in Guatemala is bordering areas with high densities of pigs not only within the country but also in El Salvador. This warrants more detailed studies of the pig population in both countries as detailed information on disease distribution is sparse in Guatemala and completely lacking from El Salvador. High concentration of pigs was also estimated in Haiti and the Dominican Republic, but no reports of detailed distribution of porcine cysticercosis could be found. Studies investigating the disease status on the island of Hispaniola are urgently needed. Porcine cysticercosis could not be confirmed in Belize, despite reports of human cysticercosis. Pigs in the southern part of Belize are known to be kept in free-range production systems. As porcine cysticercosis is endemic in both neighbouring countries not far from the border, surveys investigating the status in Belize are highly warranted.

The presence of porcine cysticercosis is a significant indicator of active transmission of *T. solium* since pigs within production systems have relatively short lifespans, become infected early in life [23], and generally do not move across large distances geographically [24, 25]. Humans are however extremely mobile and therefore establishing where cysticercosis or taeniosis was contracted is difficult. Cysticercosis in humans is further complicated by the late-onset of symptoms if any at all [26], and the difficulty in establishing a correct diagnosis in general [27]. Likewise for taeniosis, as the infection is often asymptomatic and the lifespan of the parasite unknown, establishing time and place of transmission can be difficult. Efforts are not always made to differentiate between species and reports of taeniosis could therefore often be *T. solium*, *T. saginata*, or even *T. asiatica*.

The presence of *T. solium* in Central America and the Caribbean basin identified in this study (Fig. 2) is comparable with the recently updated global endemicity map published by WHO, although WHO did not identify Guyana to be endemic [28]. Identifying endemic countries should have high priority, and national distribution maps identifying affected areas at the level of detail, such as second-level administrative level, suitable for government control programme implementation are essential for all endemic countries. Common for all countries within Central America and the Caribbean basin is that no detailed distribution maps exist. Such maps should, for control purposes, focus on the distribution of porcine cysticercosis and the degree of endemicity.

Reports from the Caribbean islands are sparse. Kurup & Hunjan [29] reported a case of *T. solium* taeniosis on Saint Lucia, but no information was included on how this case was confirmed to species level and whether the patient was suspected of having been infected on the island or abroad. Despite the lack of reports from other Caribbean islands, with the exception of Hispanola, *T. solium* should not be dismissed as a health risk. This health risk within non-endemic countries was illustrated by imported human cases of neurocysticercosis on Guadeloupe [30]. This report further supported the theory that *T. solium* is present in Haiti and the Dominican Republic as the patients originated from these countries. Further data on the presence and detailed distribution of porcine cysticercosis in Haiti and the Dominican Republic are highly warranted, as although no reports of porcine cysticercosis were found for these two countries, there is no indication that surveys have been conducted.

## Conclusion

Due to the lack of epidemiological data within Central America and the Caribbean basin, cysticercosis could be undiagnosed and underreported, and a major unrecognised health and agricultural problem in the region. More detailed information on the distribution of *T. solium* and accurate burden estimations are urgently needed from endemic areas to grasp the true extent of this zoonotic parasite and the public health and agricultural problems it potentially poses. This will allow local governments to make informed decisions on whether or not control of *T. solium* should be a priority within their respective country.

## Additional file

Additional file 1:Table S1. Countries, territories, and institute contacts. (XLSX 11 kb)
